# Interactions among Amazon land use, forests and climate: prospects for a near-term forest tipping point

**DOI:** 10.1098/rstb.2007.0036

**Published:** 2008-02-11

**Authors:** Daniel C Nepstad, Claudia M Stickler, Britaldo Soares- Filho, Frank Merry

**Affiliations:** 1 Woods Hole Research Center149 Woods Hole Road, Falmouth, MA 02540, USA; 2 Instituto de Pesquisa Ambiental da AmazôniaAvenida Nazaré, 669, CEP: 66035-170, Belém, Pará, Brazil; 3 School of Natural Resources and Environment, University of FloridaGainesville, FL 32611-6455, USA; 4 Centro de Sensoriamento Remoto, Universidade Federal de Minas GeraisCEP: 31270-901, Belo Horizonte, Minas Gerais, Brazil

**Keywords:** fire, deforestation, biofuel, feedbacks, globalization, global warming

## Abstract

Some model experiments predict a large-scale substitution of Amazon forest by savannah-like vegetation by the end of the twenty-first century. Expanding global demands for biofuels and grains, positive feedbacks in the Amazon forest fire regime and drought may drive a faster process of forest degradation that could lead to a near-term forest dieback. Rising worldwide demands for biofuel and meat are creating powerful new incentives for agro-industrial expansion into Amazon forest regions. Forest fires, drought and logging increase susceptibility to further burning while deforestation and smoke can inhibit rainfall, exacerbating fire risk. If sea surface temperature anomalies (such as El Niño episodes) and associated Amazon droughts of the last decade continue into the future, approximately 55% of the forests of the Amazon will be cleared, logged, damaged by drought or burned over the next 20 years, emitting 15–26 Pg of carbon to the atmosphere. Several important trends could prevent a near-term dieback. As fire-sensitive investments accumulate in the landscape, property holders use less fire and invest more in fire control. Commodity markets are demanding higher environmental performance from farmers and cattle ranchers. Protected areas have been established in the pathway of expanding agricultural frontiers. Finally, emerging carbon market incentives for reductions in deforestation could support these trends.

## 1. Introduction

The future of the Amazon Basin is a topic of great concern worldwide. The trees of the Amazon contain 90–140 billion tons of carbon (Soares-Filho *et al.* 2006), equivalent to approximately 9–14 decades of current global, annual, human-induced carbon emissions (Canadell *et al.* 2007). The prospect of reducing global warming and keeping global average temperatures from rising no more than 2°C will be very difficult if emissions of carbon from tropical forests worldwide, and the Amazon in particular, are not curtailed sharply in the coming years (Gullison *et al.* 2007). Beyond its role as a giant, somewhat leaky reservoir of carbon, the Amazon is home to one out of every five mammal, fish, bird and tree species in the world. Less recognized, perhaps, is the role of the Amazon in the global energy and water balance. Approximately eight trillion tons of water evaporate from Amazon forests each year, with important influences on global atmospheric circulation (IPCC 2007). The remainder of the rainfall entering this enormous basin flows into the Atlantic Ocean—15–20% of the worldwide continental freshwater run-off to the oceans.

These ecological services may be threatened by global warming through a late-century, climate-driven substitution of forests by savannah and semi-arid vegetation in what has been called the Amazon forest ‘die back’ (Nobre *et al.* 1991; Cox *et al.* 2000, 2004; Botta & Foley 2002; Oyama & Nobre 2003). However, these climate–vegetation simulations do not include land-use change, or the synergistic effects of land-use change and near-term *regional* climate change on the Amazon fire regime. Could accelerating, forest-substituting and forest-damaging economic activities interact with regional climate change to replace or degrade a large portion of the Amazon forest over the next two decades? And what counteracting trends could prevent such a dieback? These questions are the focus of this paper. We review current trends in Amazon economic, ecological and climatic processes, the growing evidence of positive feedbacks among these processes and the potential for these interactions to push Amazon forests towards a tipping point. We conclude with a brief review of some of the processes that could contribute to a strategy to reduce the chances of a major Amazon forest dieback. This synthesis draws on the published literature, some unpublished data, and presents two new maps of the Amazon.

## 2. The growing profitability of deforestation-dependent land uses

There are several trends underway that point to growing, sustained economic pressure to convert Amazon forests to crop fields and cattle pasture. First, large areas of southern and eastern Amazonia have eradicated foot-and-mouth disease, opening much of the region's cattle and swine industries to export out of the Amazon, often for higher prices (Kaimowitz *et al.* 2004; Arima *et al.* 2005; Nepstad *et al.* 2006*a*). Second, the rising international demand for agro-industrial commodities, discussed below, is colliding with the scarcity of appropriate land for agro-industrial expansion in the USA, Western Europe, China and many other agricultural countries (Brown 2004). As a result, much of the recent surge in global cropland area expansion is taking place in the Brazilian *Cerrado* and Amazon regions (Brown 2004; Shean 2004; Nepstad *et al.* 2006*a*). Third, the rising price of oil has triggered new national policy initiatives in the USA, the European Union and Brazil that feature the expansion of biofuel as a substitute for gasoline and diesel (Yacobucci & Schnepf 2007). Brazilian sugar cane ethanol will supply much of the growing global demand for ethanol because it is one of the world's most efficient and inexpensive forms of ethanol (Pimentel & Patzek 2005; World Watch 2006; Xavier 2007), and it has the greatest potential for expanded production. Similarly, palm oil is one of the most efficient sources of bio-diesel with large potential for expansion in the wetter regions of the Amazon. Finally, crop breeding programmes in Brazil have produced varieties of soya and other crops that are tolerant of the high temperatures and humidity of the Amazon region (Cattaneo 2008). These international commodity trends influence Amazon land use through linkages that we refer to as ‘economic teleconnections’ (Nepstad *et al.* 2006*a*).

We illustrate recent trends in economic teleconnections with the case of corn and sugar cane ethanol in the USA and Brazil, respectively (figure 1). With the US plan to supply 15% of its gasoline consumption through alternative fuels, including ethanol, by the year 2017 (US White House 2007), its subsidization of ethanol made from corn ($0.51 per gallon) and its ongoing tariff on imported Brazilian ethanol ($0.55 per gallon), corn prices and production are expanding (Severinghaus 2005; USDA 2006; Valle 2007; Wald & Barrionuevo 2007). Corn's expansion has displaced US soya bean production areas, pushing soya prices up by reducing the global supply of soya. Meanwhile, the expansion of sugar cane production in southern Brazil has had a similar effect, displacing soya production areas and further increasing soya prices (Valle 2007). Demands for soya are also increasing owing to the emerging Brazilian bio-diesel industry and growing imports of soya by the burgeoning animal ration industries (Fearnside 2001; Brown 2004; Clay 2004; Nepstad *et al.* 2006*a*; USDA 2007). China is the leader of a group of countries (referred to here as the ‘emerging meat-eating nations’) whose growing affluent classes are consuming larger amounts of ration-fed poultry, pork and other types of meat (Brown 2004). Soya is the best source of vegetable protein in animal ration (Mosse 1990) and is highly sought after by the animal ration industries (Brookes 2001; FAO 2004). The price of soya today is approaching its highest level in history (Zafalon 2007); as a result, the Brazilian soya harvest (2007–2008) is expected to expand 7% over the previous year (Zafalon 2007).

Growing global demands for biofuel and animal ration provide new incentives to clear forest that are already colliding with a decade-long expansion of the Amazon cattle herd. Although some soya expansion takes place through the direct conversion of forest to soya, most of the expansion is onto areas that were previously cattle pasture (Morton *et al.* 2006), pushing up land prices in the process and capitalizing ranchers who can move on to acquire land holdings deeper into the Amazon forest region. This is particularly important given the prevalence of cattle ranching as a land use and as a driver of deforestation in the region. In the Brazilian North region (including the Amazon states of Rondônia, Acre, Amazonas, Roraima, Pará, Tocantins and Amapá), the 74 million head of cattle occupy 84% of the total area under agricultural and livestock uses (IBGE 2005) and have expanded 9% yr^−1^, on average, over the last 10 years causing 70–80% of deforestation (Kaimowitz *et al.* 2004; Nepstad *et al.* 2006*a*). Hence, the expansion of soya and agro-industry generally must be viewed as a process that is, for the most part, displacing and capitalizing cattle ranching interests (Nepstad *et al.* 2006*a*). A similar process of commodity market-driven soya expansion is underway in the Bolivian Amazon (Hecht 2005). These trends will grow stronger if the Brazilian Real (currently at R$1.75 per dollar) weakens. It is currently at its strongest level since 1998 (Nepstad *et al.* 2006*a*).

## 3. Forest degradation through drought, fire, fragmentation and logging

The globalization of deforestation in some parts of the Amazon is superimposed upon synergistic processes of forest degradation. Positive feedbacks among drought, forest fire and economic activities hold the potential to degrade large areas of Amazon forest over the coming years. The potential is best understood by first considering the limits of Amazon forest drought tolerance. Nearly half of the forests of the Amazon maintain full leaf canopies during dry seasons that range from three to five months, indicating a high tolerance of drought (Nepstad *et al.* 1994; Myneni *et al.* 2007; Saleska *et al.* 2007). These forests have many trees with deep roots and are able to avoid severe drought stress by absorbing moisture stored deep in the soil, even as surface soil moisture supplies are depleted (Nepstad *et al.* 1994, 2004, 2007*a*; Hodnett *et al.* 1995; Bruno *et al.* 2006). In a partial rainfall exclusion experiment, it was found that the trees in a central Amazon forest avoided drought by absorbing deep soil moisture, but they also delayed the onset of drought through hydraulic redistribution of soil moisture (Oliveira *et al.* 2005) and foliar uptake of dry season rainwater and dew (Cardinot 2007). However, Amazon forest drought tolerance has its limits. In this same experiment, a drought threshold was reached beyond which the mortality of large (above 30 cm in stem diameter) trees rapidly climbed sixfold (Nepstad *et al.* 2007*a*; Brando *et al.* 2008). This threshold was reached by experimentally reducing rainfall by only one-third over a 2.5-year period, lowering plant-available soil water (PAW) to below 30% of its maximum value. The El Niño episode of 1997–1998 may have brought some Amazon forests close to this drought threshold (Nepstad *et al.* 1999, 2004); in the central Amazon, tree mortality increased 50% following this drought (Williamson *et al.* 2000).

The drought-induced death of Amazon forest's dominant organisms (its canopy trees) may increase the prospect of further disturbance from fire for years afterwards (Holdsworth & Uhl 1997; Brando *et al.* 2008). The leaf crowns of canopy trees separate the zone of solar radiation absorption and conversion to sensible heat high above the ground from the dark, humid forest floor far below. Each canopy tree that dies creates a canopy gap through which radiant energy penetrates into the forest, warming the forest interior. Field studies involving experimental forest fires indicate that forest susceptibility to fire is inversely related to the density and average height of the leaf canopy (Ray *et al.* 2005).

Land-use activities of the Amazon contribute to forest susceptibility to fire by providing ignition sources, by fragmenting the forest, and by thinning the forest through logging. Although ignition from lightening is rare in the central forests of the Amazon, man-made sources of ignition are increasingly abundant. Fires set to burn felled forests in preparation for crops or pasture, or to improve pasture forage, frequently escape beyond their intended boundaries into neighbouring forests. During the severe drought of 1998, approximately 39 000 km^2^ of standing forest caught fire in the Brazilian Amazon (Alencar *et al.* 2006), which is twice the area of forest that was clear-cut that year. During the severe drought of 2005 (Aragão *et al.* 2007), at least 3000 km^2^ of standing forest burned in the southwest Amazon (Brown *et al.* 2006). These low, slow-moving fires can be deceptively destructive, killing from 10 to 50% of trees (above 10 cm in diameter; Cochrane & Schulze 1999; Barlow & Peres 2004; Alencar *et al.* 2006). Forest fire can therefore increase susceptibility to further burning in a positive feedback by killing trees, opening the canopy and increasing solar penetration to the forest floor (Nepstad *et al.* 1995, 1999, 2001; Cochrane & Schulze 1999; Cochrane *et al.* 1999; Alencar *et al.* 2004).

Forest degradation is also fostered by fragmentation and edge effects associated with forest clear-cutting for pasture formation. Tree mortality and forest flammability are higher along forest edges (Laurance *et al.* 1997; Alencar *et al.* 2004). Selective logging, which can damage up to 50% of the leaf canopy (Uhl & Vieira 1989), also increases forest susceptibility to fire (Uhl & Kauffman 1990; Holdsworth & Uhl 1997). Selective logging degrades about as much forest each year as does clear-cutting (Nepstad *et al.* 1999; Asner *et al.* 2005).

Tree mortality induced by drought, fire, fragmentation and selective logging is the initial step of a process of forest degradation that is reinforced by positive feedbacks that can, potentially, convert forest ecosystems into fire-prone ‘brush’ vegetation (figure 2). Although we are unaware of systematic field surveys of repeatedly burned forests to document the types and extent of this severe forest degradation, our field observations in northeastern Mato Grosso, southeastern and eastern Pará states and near Santarém suggest that repeated disturbance can transform Amazon forests into low-stature ecosystems dominated by invasive, coppicing tree species (such as *Solanum crinitum* and *Vismia guianensis*), grasses (including *Imperata brasiliensis*, *Paspalum* spp.), bamboo (*Guandu* spp.) and ferns (such as *Pteridium aquilinum*; Ray *et al.* 2005; Alencar *et al.* 2006; D. C. Nepstad 2004–2007, unpublished data). Although still rare in occurrence, forests invaded by grasses may be the most susceptible to conversion to brush owing to the large amount of fuel produced by many graminoids (Uhl & Kaufmann 1990) and owing to their inhibitory effect on tree regeneration (Nepstad *et al.* 1996). Grasses invade forests through either seed dispersal by animals or wind, or deliberate seeding by cattle ranchers seeking to capitalize on the burning of the forest reserves on their properties (D. C. Nepstad 2007, unpublished data). The potential of grasses to displace extensive areas of forest is best exemplified in Southeast Asia, where a single grass species (*Imperata cylindrica*) now dominates soils once covered by moist tropical forest across approximately 300 000 km^2^ (MacDonald 2004).

Amazon forest susceptibility to degradation may vary spatially along edaphic, climatic, vegetational and economic gradients, but these gradients are poorly understood. Current evidence suggests a few key features of Amazon forest vulnerability to degradation that could be tested through further research. Forest degradation may be most probable when (i) high levels of tree mortality are induced by drought, fire, fragmentation or logging, (ii) propagules of high-fuel grasses and, perhaps, ferns or bamboo are abundant following tree mortality, (iii) ignition sources are present, and (iv) the forest is subjected to severe seasonal or episodic drought. The portion of the Amazon under which these conditions are all met may be expanding.

## 4. The drying of the Amazon

Several lines of evidence suggest that the eastern Amazon may become drier in the future, and that this drying could be exacerbated by positive feedbacks with the vegetation. At the broadest temporal and spatial scale, most global circulation models (GCMs) predict that greenhouse gas accumulation and associated increases in the radiative forcing of the atmosphere will cause a substantial (more than 20%) decline in rainfall in eastern Amazonia by the end of the century, with the biggest decline occurring in the dry season (IPCC 2007; Malhi *et al.* 2008). When GCMs are coupled to dynamic vegetation models, some predict a large-scale, late-century substitution of closed-canopy evergreen forest by savannah-like and semi-arid vegetation, mostly in the eastern end of the basin (Cox *et al.* 2000, 2004; Botta & Foley 2002; Oyama & Nobre 2003). The lower evapotranspiration and higher albedo of this new vegetation reinforces the drying in a positive feedback. Most coupled climate–vegetation models, however, do not predict this dieback (Friedlingstein *et al.* 2003; Gullison *et al.* 2007). None of these models have incorporated the positive feedbacks between land use, forest fire and drought that have been described for the Amazon region (Nepstad *et al.* 1995, 1999, 2001; Cochrane *et al.* 1999), however, and therefore may be conservative.

A more likely near-term shift in Amazon climate may be associated with changes in sea surface temperature that are usually associated with Amazon drought. Rainfall tends to decline in the Amazon when sea surface temperatures rise along the Pacific coast of northern South America through El Niño episodes (Marengo *et al.* in press). The warming of the sea surface between western Africa and the Gulf of Mexico, the Northern Tropical Atlantic Anomaly, is also associated with drought in the Amazon Basin (Marengo *et al.* in press). Some climatologists believe that these anomalies will become more frequent as greenhouse gases accumulate further in the atmosphere (Trenberth & Hoar 1997; Timmermann *et al.* 1999; Hansen *et al.* 2006).

Deforestation alone may also provoke reductions in rainfall (Nobre *et al.* 1991; Silva Dias *et al.* 2002; Da Silva *et al.* in press). It now appears that clearing beyond approximately 30% of the forests of the region may trigger a decline in rainfall (Sampaio *et al.* 2007; Da Silva *et al.* in press) and that projected future trends in deforestation may inhibit rainfall more during El Niño episodes than during non-El Niño years (Da Silva *et al.* in press).

On the shortest time scale, fire itself can inhibit rainfall by liberating large amounts of particulate matter into the atmosphere, providing an excess of condensation nuclei (Andreae *et al.* 2004). The scientific community still does not know how important this phenomenon is, but there is anecdotal evidence that this rainfall inhibition is already exerting an effect on farmers and ranchers. Pilots and farmers in the Xingu headwaters region of Mato Grosso claim that the rainy season begins later in the year by several weeks when the density of smoke is high (J. Carter 2007, personal communication).

## 5. Interactions among ecosystems, economics and climate: an Amazon forest tipping point

This review suggests that the economic, ecological and climatic systems of the Amazon may be interacting to move the forests of this region towards a near-term tipping point. In this scenario, the growing profitability of deforestation-dependent agriculture and cattle ranching provides an expanding frontier of forest fragmentation and ignition sources that inhibits rainfall as forests are replaced by fields and pastures and as fires fill the late dry season atmosphere with aerosols. Forests damaged by drought, logging, fragmentation and previous fire burn repeatedly as tall canopy tree species are gradually replaced by coppicing trees, grasses and other high-biomass plants. These local and regional processes are exacerbated when sea surface anomalies and extreme weather events cause severe drought episodes and the burning of vast forested landscapes. Global warming reinforces these trends by elevating air temperatures, increasing dry season severity and increasing the frequency of extreme weather events (IPCC 2007; figure 2).

We provide a preliminary map and quantitative estimate of the potential impacts of a near-term forest dieback by assuming ‘business-as-usual’ patterns of future deforestation as estimated by Soares-Filho *et al.* (2006), by assuming that Amazon climatic conditions of the January 1996 through December 2005 decade (Nepstad *et al.* 2004) are repeated in the future, that a tree mortality threshold is exceeded when PAW falls below 30% of its maximum value to a depth of 10 m (Nepstad *et al.* 2004, 2007*a*), and that logging will expand across the Amazon as described in the rent-based economic model of Merry *et al.* (in press) (figure 3). We do not invoke fire in this estimate, although it is probable that a substantial portion of the forests that are damaged by drought and/or logging, and additional forests that are not damaged, will experience understorey fire. Given these assumptions, 31% of the Amazon closed-canopy forest formation will be deforested and 24% will be damaged by drought or logging by the year 2030. If we assume that rainfall declines 10% in the future, then an additional 4% of the forests will be damaged by drought. If we assume that carbon release from deforestation is as described in Soares-Filho *et al.* (2006), that selective logging reduces forest carbon stocks by 15% (Asner *et al.* 2005), that drought damage causes a 10% reduction in forest biomass (Nepstad *et al.* 2007*a*) and that fire affects 20% of the forests damaged by drought or logging, releasing an additional 20% of forest carbon to the atmosphere, then 15–26 Pg of carbon contained in Amazon forest trees will be released to the atmosphere in less than three decades in a large-scale dieback without invoking the influence of global warming. This is equivalent to 1.5–2.6 years of current, worldwide, average, human-induced carbon emissions (Canadell *et al.* 2007).

There is reason to believe that this scenario is conservative. For example, high temperatures and wind speeds and a prolonged dry season in northeastern Mato Grosso state in 2007 provoked extensive forest fires in an important example of the potential for severe weather events to rapidly accelerate the forest degradation process (D. C. Nepstad *et al*. 2007, unpublished data), much as they did in 1998 and 2005 (Alencar *et al.* 2006; Brown *et al.* 2006).

## 6. Avoiding the Amazon tipping point

Several important trends hold the potential to prevent or, at least, postpone a large-scale forest dieback and provide some elements of a comprehensive Amazon conservation strategy. First is the tendency of landholders to avoid the use of fire as a land management tool and to invest more in the prevention of accidental fire as they accumulate fire-sensitive investments on their properties (Nepstad *et al.* 2001; Bowman *et al.* in press). As long as cattle ranchers and farmers believe that their investments in orchards, tree plantations, improved forage grass and timber management will be lost to fire, they are less likely to make these investments and more likely to continue their extensive cattle ranching or swidden agriculture. However, as these fire-sensitive investments accumulate in the landscape, a tipping point may be reached beyond which the many landholders who have made these investments convince their fire-using neighbours to use fire more judiciously.

Second is the change in landholder behaviour that is already evident in places in Mato Grosso and that could rapidly become widespread when sound land stewardship and compliance with environmental legislation are viewed by a growing number of producers as the necessary conditions for participating in commodity markets and for obtaining access to credit and financing. The soya growers of Mato Grosso are in the midst of such a change today as they enter their second year of a moratorium imposed by the Associação Brasileira das Indústrias de Óleos Vegetais (The Brazilian Vegetable Oil Industry Associaton). This 2-year moratorium on the purchase of soya planted on soils recently cleared from Amazon forest was stimulated by a campaign against Amazon soya initiated by the environmental organization Greenpeace (Greenpeace 2006). Soya producers and their organizations are currently developing criteria systems that would certify their farms as environmentally sound (Arini 2007). It is in the context of market pressures and certification systems that landholders are agreeing to adopt fire prevention techniques such as fire breaks along their forest borders, to seek compliance with the private forest reserve requirements of federal legislation and to conserve their riparian zones.

Third is the prospect of restricting the advance of Amazon cattle ranching. Approximately one-quarter of the previously forested lands in the Brazilian Amazon are in some stage of abandonment (Houghton *et al.* 2000), and most of these are degraded cattle pastures. The replacement of carbon- and species-rich forests with cattle production systems that support less than one animal unit per hectare presents very high societal costs and low societal benefits, thereby violating Brazil's own constitution, which states that the ‘social function’ of the land is to provide maximum economic and ecological benefits to the society (Benatti 2003; Ankersen & Ruppert 2006). One way to reduce deforestation driven by cattle ranchers, many of whom are using pastures to help stake claims to land parcels (Alston *et al.* 1999; Alencar *et al.* 2004), would be to prohibit the clearing of lands that are marginally suitable for mechanized agriculture (except where smallholder agriculture systems are appropriate). In an initial attempt to map the areas that are the most suitable for highly productive, mechanized agriculture, we employed the Shuttle Radar Topography Mission (Farr *et al.* 2007) dataset to map slope and height above river channels (as a measure of potential for inundation), and the RADAMBRASIL and FAO soils maps (described in Nepstad *et al.* 2004) to identify soils that typically contain rocks, impeding layers, and poor drainage (ultisols, lithosols, sands and hydromorphic soils; figure 4). The resulting map correctly identifies large areas of arable land where the majority of Amazon soya production is located, in northern Mato Grosso and in the Santa Cruz region of Bolivia. It also indicates potential for agro-industrial expansion in southern Peru, in the Madre de Dios region. These regions have strong dry seasons and may be suitable for soya bean expansion and, perhaps, other crops that require a dry period. A large block of arable, forested land with very little rainfall seasonality is also found in northern Colombia, northwestern Amazonas state and Northern Peru. This area may be the target of oil palm expansion, which needs little rainfall seasonality. The total area of the Amazon that is ‘suitable’ for industrial agriculture (with no edaphic or climatic restrictions) is 33%, while the area that is ‘very suitable’ (with only one restriction) is 22%. Constraining cattle pasture expansion in the Amazon would not necessarily restrict the growth rate of the Amazon cattle herd. Cattle production systems that incorporate silage and agricultural residue into the diet of the herd can achieve grazing densities of more than six animals per hectare (Landers *et al.* 2005; M. Reis *et al*. 2007, personal communication), and will be favoured if land prices escalate through growing demands for Amazon soil.

The prospect of restricting the expansion of agricultural and livestock production in the Amazon to those lands of high suitability becomes plausible in the light of a fourth important trend. Most parks and biological reserves in the Amazon have been created far from the agricultural frontier, where the opportunity costs of conservation are low and where, unfortunately, these protected areas exert little influence on deforestation (Nepstad *et al.* 2006*b*). But this historical trend may be changing. From early 2004 to 2006, 23 Mha of protected areas and national forest districts were created in the Brazilian Amazon, including large areas of protected land in the pathway of the expanding agricultural frontier (Campos & Nepstad 2006). This historic achievement was made possible by a participatory, multiple-stakeholder, regional planning process that has emerged along the BR-163 (Santarém-Cuiabá) highway, which is slated for paving, and by the smallholder farmers’ movement of the Transamazon highway region, which successfully initiated the ‘Terra do Meio’ conservation mosaic (Campos & Nepstad 2006). Through the combination of (i) regional, participatory planning processes that lead to effective land-use zoning systems, (ii) recent government demonstrations of political will in establishing protected areas expressed through the Amazon region protected area programme and in cracking down on corrupt government employees, and (iii) growing market demands for higher environmental and social performance of Amazon farmers and ranchers, it is conceivable that deforestation could be restricted to the one-quarter of the region that is suitable for modern mechanized production (figure 4).

A fifth potentially robust mechanism for counteracting the pressures to clear and degrade Amazon forest, and that could reinforce efforts to constrain the expansion of cattle ranching, is emerging within the United Nations Framework Convention on Climate Change (UNFCCC) negotiations. The proposal to compensate those tropical nations that succeed in reducing their nationwide emissions of greenhouse gases from deforestation (Santilli *et al.* 2005) has gained momentum in the UNFCCC process (Gullison *et al.* 2007), and it could provide an important economic incentive for countries and, in turn, landholders who reduce those land-use activities that release greenhouse gases to the atmosphere. Negotiation of this post-2012 component of the UNFCCC will be completed by the end of 2009. Since most of the Amazon carbon emissions are caused by forest conversion to cattle ranching with low profitability (Arima & Uhl 1997; Kaimowitz *et al.* 2004; Margulis 2003), such a mechanism could restrict the expansion of this land use to the remaining forest regions of the Amazon, thus reducing the risk of a near-term forest dieback scenario. We estimate that deforestation in the Brazilian Amazon could be brought to approximately zero within 10 years within the context of a 30-year programme costing $8 billion, or less than $2 per tonne of reduced carbon emission (Nepstad *et al.* 2007*b*).

## 7. Conclusion

Synergistic trends in Amazon economies, forests and climate could lead to the replacement or severe degradation of more than half of the closed-canopy forests of the Amazon Basin by the year 2030, even without invoking fire or global warming. Counteracting these trends are emerging changes in landholder behaviour, recent successes in establishing large blocks of protected areas in active agricultural frontiers and practical techniques for concentrating livestock production on smaller areas of land that could reduce the likelihood of a large-scale self-reinforcing replacement of forest by fire-prone brush. In the long term, however, the avoidance of an Amazon forest dieback may depend upon worldwide reductions of greenhouse gas emissions that are large enough to prevent global temperatures from rising more than a degree or two.

## Figures and Tables

**Figure 1:**
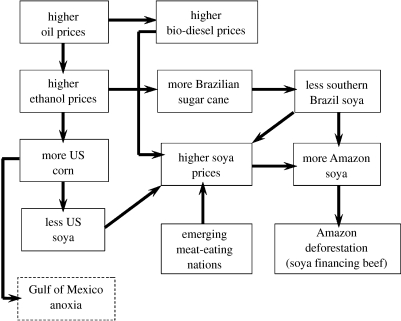
Economic teleconnections between US investments in corn-based ethanol production, Brazilian investments in sugar cane-based ethanol production and Amazon deforestation.

**Figure 2:**
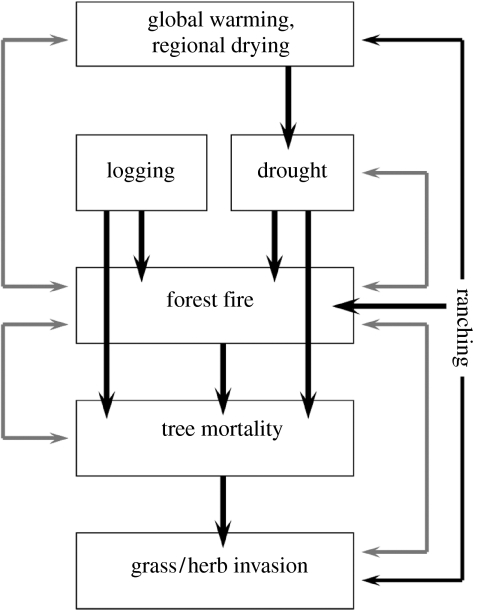
Diagram of the processes and interactions that could lead to a near-term Amazon forest dieback.

**Figure 3:**
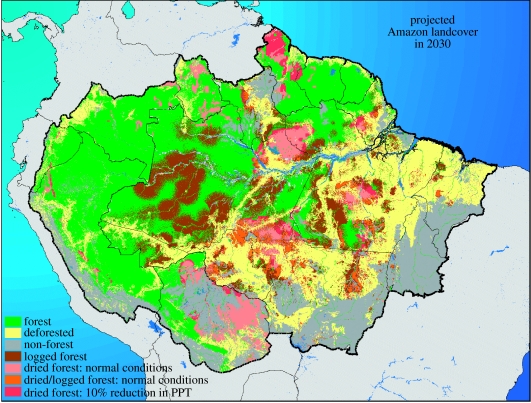
A map of Amazonia 2030, showing drought-damaged, logged and cleared forests assuming the last 10 years of climate are repeated in the future. See text for further details. PPT, precipitation.

**Figure 4:**
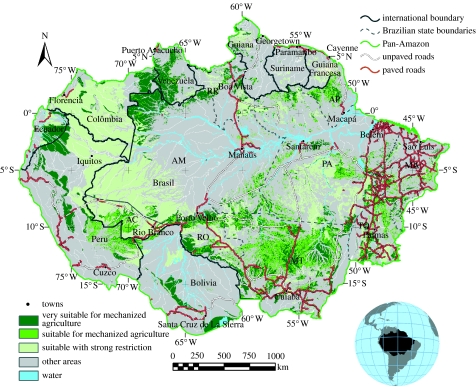
Soil suitability map for mechanized agriculture in the Pan-Amazon region. Restrictions include slope (more than 2%), inundation risk and poor soil (ultisols, hydromorphic soils, sands and lithosols). See text for further methodological details. State abbreviations: AM, Amazonas; RR, Roraima; AP, Amapá; PA, Pará; TO, Tocantins; MT, Mato Grosso; RO, Rondônia; AC, Acre; MA, Maranhão.
